# Nanotechnology-Enabled Strategies to Overcome Antibiotic Resistance in Respiratory Infections: Mechanisms, Platforms, and Translational Challenges

**DOI:** 10.3390/biomedicines14081693

**Published:** 2026-07-28

**Authors:** Ghazala Muteeb, Rayan A. Siraj

**Affiliations:** 1 Department of Nursing, College of Applied Medical Science, King Faisal University, Al-Ahsa 31982, Saudi Arabia; 2 Department of Respiratory Therapy, College of Applied Medical Sciences, King Faisal University, Al-Ahsa 31982, Saudi Arabia; rsiraj@kfu.edu.sa

**Keywords:** antimicrobial resistance, nanocarriers, nanotechnology, pulmonary drug delivery, respiratory infections

## Abstract

Antimicrobial resistance (AMR) in respiratory infections represents a major global health challenge, compounded by biological barriers that limit the effectiveness of conventional antibiotics, including mucus hypersecretion, biofilm formation, and intracellular pathogen persistence. Nanotechnology has emerged as a promising platform for addressing these limitations through advanced drug-delivery strategies. This narrative review provides an integrated overview of nanocarrier systems—including lipid-based (e.g., liposomes, solid lipid nanoparticles), polymeric (e.g., PLGA, chitosan), and inorganic nanoparticles (e.g., silver, gold, zinc oxide)—with emphasis on their pharmaceutical design parameters for pulmonary delivery. Key mechanisms by which nanotechnology enhances antimicrobial efficacy include targeted and controlled drug delivery, improved penetration of mucus and biofilms via surface engineering, synergistic combination therapies, and intrinsic antimicrobial activity through mechanisms such as reactive oxygen species generation. Preclinical studies targeting major respiratory pathogens, including *Pseudomonas aeruginosa*, *Mycobacterium tuberculosis*, *Streptococcus pneumoniae*, and methicillin-resistant *Staphylococcus aureus*, demonstrate enhanced biofilm disruption, intracellular drug delivery, and reductions in bacterial burden. However, important translational challenges remain, including long-term safety, manufacturing scalability, regulatory complexity, and the potential for microbial adaptation. Future directions focus on stimuli-responsive systems, inhalable formulations, and biomimetic platforms to improve targeting and therapeutic precision. Collectively, nanotechnology represents a delivery-oriented strategy with the potential to enhance existing antimicrobial therapies and support the development of more effective interventions against resistant respiratory infections.

## 1. Introduction

Antimicrobial resistance (AMR) refers to the capacity of microorganisms to acquire resistance against antibiotics and other antimicrobial agents. The AMR crisis is one of the most serious threats to contemporary medicine worldwide. In 2019, 1.27 million deaths were directly related to bacterial AMR, and it contributes to 4.95 million fatalities across the globe in low- and middle-income countries [1]. According to reports, without effective intervention, AMR will claim 10 million lives in a year by 2050, which is more than cancer, leading to cumulative economic damage of 100 trillion dollars [2]. The World Health Organisation (WHO) has categorised AMR as one of the top ten global threats to public health, largely due to the proliferation of resistance genes and the emergence of multidrug-resistant (MDR) and extensively drug-resistant (XDR) pathogens. The WHO has identified highly virulent bacteria and compiled a list of pathogens resistant to multiple antibiotics. ESKAPE pathogens, namely, *Enterococcus faecium*, *Staphylococcus aureus*, *Klebsiella pneumoniae*, *Acinetobacter baumannii*, *Pseudomonas aeruginosa*, and *Enterobacter* species, are believed to contribute to the majority of hospital-acquired infections [3].

WHO has also curated the AWaRe (Access, Watch, Reserve) classification of antibiotics based on their clinical and resistance significance, which serves as a tool for policymakers, researchers, and healthcare providers to better support antibiotic prescribing, stewardship activities, and monitoring. It includes antibiotics such as amoxicillin and cefazolin in the access category, Azithromycin and Cefixime in the watch category, and Colistin and Linezolid in the reserve category [4]. Alarmingly, resistance rates for these critical agents exceed 50% in some regions, rendering previously treatable infections potentially fatal. The world’s antibiotic pipeline remains severely limited; since the 1980s, there has been a stall in the discovery of new antibiotic classes for clinical use [5]. Although the development of new antibiotics is critically important, it alone is not enough to combat AMR. New molecules are likely to encounter the same resistance mechanisms and solubility issues that have crippled current treatments, and the financial incentive to develop antibiotics is lacking, with many pharmaceutical firms leaving the industry. This is an important gap that must be addressed by considering an alternative strategy: advanced drug delivery systems, capable of improving the performance of current antibiotics and providing completely new therapeutic modalities [6].

Respiratory tract infections are among the most clinically important manifestations of antimicrobial resistance (AMR). Despite significant decreases in child mortality over the past few decades, LRI is still the largest cause of mortality in children globally (Global Burden of Disease (GBD) 2021 study [7]). Conventional antibiotic therapy for community- and hospital-acquired respiratory infections caused by major bacterial pathogens such as *Streptococcus pneumoniae*, *Klebsiella pneumoniae*, *Pseudomonas aeruginosa*, *Acinetobacter baumannii*, and *Staphylococcus aureus* is becoming less effective due to the emergence of multidrug-resistant (MDR) and XDR phenotypes [8,9].

AMR consequences extend beyond clinical outcomes; they also impose high economic costs on healthcare systems. Respiratory infections that are resistant to antibiotics have been linked to longer hospitalizations, more admissions to the intensive care unit, higher antibiotic usage, and higher health care costs. Such problems have been driving research into new methods of drug delivery, in addition to the discovery of new antimicrobial agents. Nanotechnology-based drug delivery systems have been shown to be a promising platform that can overcome several limitations of conventional antimicrobial therapy by improving pulmonary drug deposition and penetration into mucus and biofilms, reducing systemic toxicity, and enhancing intracellular antibiotic delivery [10].

### 1.1. Why Respiratory Infections Are Particularly Vulnerable to Resistance

Respiratory tract infections (RTIs) represent a uniquely vulnerable niche in the AMR crisis. Bacterial pneumonia remains the leading infectious cause of death in children worldwide, accounting for 15% of all deaths in children under five years old [11]. In adults, lower respiratory tract infections are the leading cause of death in low-income countries, with healthcare costs exceeding $15 billion annually in the United States alone [12,13]. The pulmonary environment presents specific anatomical and physiological barriers that profoundly compromise antibiotic efficacy:

***The Protective Barrier Function of Respiratory Mucus.*** The lung’s epithelial surface is lined with a continuous layer of respiratory mucus—a viscoelastic gel composed of mucin glycoproteins, water, lipids, and antimicrobial peptides. Under normal conditions, this mucus layer serves as the first line of defence, trapping inhaled pathogens for clearance by the mucociliary escalator [14]. However, in respiratory infections, mucus hypersecretion and altered rheology (thickened, dehydrated mucus) create a formidable barrier that impedes antibiotic penetration. Many antibiotics, particularly aminoglycosides and macrolides, bind to mucins, reducing free drug concentration at the epithelial surface [15]. This barrier is especially problematic in chronic respiratory diseases such as cystic fibrosis (CF), chronic obstructive pulmonary disease (COPD), and bronchiectasis, where mucus accumulation and impaired clearance perpetuate infection.

***The Role of Bacterial Biofilms in Evading Host Immunity and Antibiotics*.** Biofilms—structured communities of bacteria encased in a self-produced extracellular polymeric matrix (EPS) composed of polysaccharides, extracellular DNA, and proteins—are a hallmark of chronic respiratory infections [16]. Biofilm formation dramatically increases antibiotic resistance, with minimum inhibitory concentrations (MICs) often exceeding planktonic MICs by 100- to 1000-fold [17]. Multiple mechanisms contribute to this recalcitrance: the EPS matrix physically excludes antibiotics; nutrient gradients create metabolically dormant “persister” cells that tolerate antibiotic killing; and the biofilm microenvironment facilitates horizontal gene transfer, accelerating resistance spread [18]. In conditions such as CF, *P. aeruginosa* biofilms persist in the airways despite aggressive antibiotic therapy, leading to chronic, progressive lung damage that remains the leading cause of mortality in CF patients [19].

***The Challenge of Intracellular Pathogens.*** Several key respiratory pathogens, including *Mycobacterium tuberculosis*, *Legionella pneumophila*, and *Chlamydia pneumoniae*, reside and replicate within host cells—primarily alveolar macrophages—where they evade both the immune system and antibiotic action [20]. Poor membrane permeability, active efflux by host cell transporters, sequestration in phagolysosomes, and altered drug activity at acidic pH act as barriers to the intracellular activity of conventional antibiotics [21]. *M. tuberculosis*, the causative organism for tuberculosis (TB), has shown the ability to escape macrophages for decades, which results in prolonged multidrug therapy—6 to 9 months for drug-sensitive TB and 18–24 months for MDR-TB. This, in turn, results in poor adherence and fuels the emergence of resistance [22]. The intracellular niche thus represents both a sanctuary for pathogens and a critical vulnerability in current treatment paradigms.

***Major Antimicrobial Resistance Mechanisms in Respiratory Pathogens.*** Besides biological barriers, respiratory pathogens also have intrinsic and acquired mechanisms of resistance that make them more resistant to conventional antibiotic therapy. Biofilm formation is a key factor in chronic respiratory infections. The extracellular polymeric substance (EPS), composed of polysaccharides, proteins, extracellular DNA, and lipids, is a physical diffusion barrier that impedes antibiotic penetration and creates nutrient and oxygen gradients that favour metabolically dormant persister cells, which are less susceptible to antibiotics. In addition, horizontal gene transfer is more likely to occur in bacterial cells within biofilms, which allows for rapid spread of antimicrobial resistance determinants within the context of biofilm. These properties contribute to chronic infections in diseases such as cystic fibrosis, chronic obstructive pulmonary disease, and bronchiectasis, where *Pseudomonas aeruginosa* biofilms are difficult to eradicate [23,24]. Another important mechanism of treatment failure is intracellular persistence. Alveolar macrophages and other cells in the host environment are able to harbour pathogens such as Mycobacterium tuberculosis, Legionella pneumophila, and Chlamydia spp., which escape exposure to antibiotics because of limited permeability of the cell membrane and sequestration of the pathogen in the cell. Pathogens that are usually considered “extracellular” have also been found to temporarily reside inside host cells and evade the immune system and antimicrobial response.

Nanotechnology has emerged as a promising strategy to overcome resistance-associated barriers. Antibacterial nanoparticles can be surface-engineered to enhance their diffusion into airway mucus and penetration of the EPS matrix, thereby increasing the availability of antibiotics within the biofilm. Furthermore, nanoparticles have been developed to deliver multiple antibiotics and other agents that disrupt biofilms, such as enzymes that degrade extracellular DNA or matrix polysaccharides, and quorum-sensing inhibitors that disrupt the communication and maturation of bacterial biofilms. Targeted nanocarriers also improve the intracellular delivery of antibiotics by increasing drug uptake by infected macrophages and maintaining prolonged release at intracellular sites of infection. Together, these multifunctional approaches will optimize local drug levels, facilitate bacterial clearance, and possibly decrease the risk of recurrent and chronic respiratory infections, and maximize the therapeutic value of current antibiotics [25].

***Pharmacokinetic and Biodistribution Limitations.*** Unfavourable pharmacokinetic properties limit the efficacy of small molecules in combating respiratory infections. Off-target toxicities, particularly nephrotoxicity from aminoglycosides and vancomycin, poor penetration into lung tissue and inadequate accumulation in abscesses hinder therapeutic effectiveness [26]. For inhaled antibiotic formulations, challenges include efficient aerosolisation, uniform deposition, avoidance of macrophage clearance, and sustained retention at the site of infection [20].

### 1.2. Nanotechnology: A Paradigm-Shifting Platform

The combination of these issues—the growing resistance, the biological hindrance, and the lack of new antibiotic development—has triggered the development of nanotechnology as a revolutionary solution to the problem of AMR. The term nanotechnology, which refers to engineering materials with one or more dimensions between 1 and 100 nm, offers special properties that can be utilised to address the problem of traditional antibiotics [27]. Importantly, the relevance of nanotechnology in this context lies in its ability to directly address the biological and pharmacokinetic barriers outlined in the previous section. For example, surface-engineered nanoparticles can enhance penetration through mucus layers, while charge-modulated and enzyme-responsive systems improve diffusion within biofilms. Similarly, ligand-functionalised nanocarriers facilitate targeted delivery to intracellular pathogens within alveolar macrophages, and controlled-release formulations help maintain therapeutic drug concentrations in the lung microenvironment. These capabilities position nanotechnology as a delivery-oriented strategy rather than solely an antimicrobial innovation.

Nanomedicines can be engineered to enhance drug solubility and stability, enabling formulation of poorly water-soluble antibiotics and protecting labile drugs from degradation [28]. In addition, they prolong circulation time and control release kinetics, thereby reducing dosing frequency and maintaining therapeutic concentrations at infection sites [29]. Surface functionalisation further enables targeted delivery to pathogens via ligand functionalisation, while their nanoscale size facilitates penetration across barriers such as mucus, biofilms, and cellular membranes [30,31]. Nanocarriers also support the co-delivery of multiple therapeutic agents, enabling synergistic combinations and suppressing the emergence of resistance [32]. Beyond serving as delivery systems, certain nanomaterials exhibit intrinsic antimicrobial activity through mechanisms that offer alternative pathways to overcome conventional resistance [33].

While these properties are widely reported, their practical significance lies in their capacity to overcome specific limitations of conventional antibiotic therapy. In particular, the ability to modulate drug distribution, enhance local retention, and enable site-specific delivery may substantially improve therapeutic outcomes in respiratory infections, where conventional pharmacokinetics often fail to achieve effective drug concentrations at the site of infection.

The past 10 years have seen an explosion in the study of nanomedicine in infectious disease, and multiple formulations have achieved clinical translation. Approved for *Mycobacterium avium* complex lung disease, Liposomal amikacin inhalation (Arikayce^®^, Insmed Inc., Bridgewater, NJ, USA) has demonstrated safe and feasible delivery of nanomedicine to the lungs [34]. Many other nanocarrier systems, such as polymeric nanoparticles, solid lipid nanoparticles, and inorganic nanomaterials, are progressing in preclinical and clinical development, covering an enormous range of respiratory pathogens. However, despite this rapid expansion, the majority of these systems remain at the preclinical stage and have limited translation into routine clinical practice. This highlights an important gap between technological development and clinical applicability.

Collectively, these findings suggest that nanotechnology offers a complementary approach to conventional antimicrobial strategies by addressing delivery-related limitations that contribute to therapeutic failure. Rather than replacing existing antibiotics, nanocarrier systems may enhance their effectiveness by improving drug accessibility within the complex pulmonary environment. This conceptual shift provides the basis for exploring nanomedicine as a targeted solution for antimicrobial resistance in respiratory infections.

### 1.3. Scope and Aim of This Review

Given the rapid expansion of this field, a critical synthesis of nanotechnology-based interventions for combating antibiotic resistance in respiratory infections is timely and necessary. This narrative review will present a critical, integrated overview of current research, with particular emphasis on several key aspects. First, it examines the major classes of nanocarriers employed for pulmonary drug delivery, including their pharmaceutical design parameters and suitability for respiratory applications. It explores specific mechanisms by which nanotechnology overcomes biological barriers and resistance pathways that limit the efficacy of conventional antibiotics. Furthermore, the review highlights applications against clinically relevant respiratory pathogens, such as *P. aeruginosa*, *M. tuberculosis*, *S. pneumoniae*, and MRSA, with a focus on how nanocarrier-based strategies address pathogen-specific challenges. Finally, it discusses translational considerations, including safety, scalability, regulatory frameworks and emerging directions.

By integrating recent advances and critically evaluating the evidence, this review seeks to guide the rational design of next-generation nanomedicines and to identify priorities for future research and development. The ultimate goal is to provide a resource for researchers, clinicians, and policymakers working to harness the transformative potential of nanotechnology in the global fight against antibiotic-resistant respiratory infections.

## 2. Body of the Review

### 2.1. Types of Nanocarriers for Respiratory Drug Delivery

Rational design of nanocarriers for pulmonary applications must carefully consider physicochemical characteristics that determine aerosolisation, lung deposition, mucus penetration, cellular uptake, and, ultimately, treatment efficacy. Particle size and size distribution (polydispersity index, PDI), surface charge (zeta potential), drug-loading capacity, encapsulation efficiency, release kinetics and biocompatibility are crucial pharmaceutical quality parameters [29]. In respiratory delivery, nanoparticles preferably have hydrodynamic diameters of 50–200 nm—small enough to prevent quick macrophage clearance, but large enough to escape exhalation and obtain peripheral lung deposition [35]. Surface charge affects the mucus penetration (neutral or PEGylated particles have an easier time penetrating the mucus) and the uptake by the cellular system (cationic particles have a higher chance of internalising but can also be more toxic) [36]. Lipid-based systems, polymeric nanoparticles, and inorganic nanomaterials are the major classes of nanocarriers studied in respiratory drug delivery. These nanocarrier systems differ significantly in their physicochemical properties, mechanisms of action, and translational potential. While lipid-based systems are generally favoured for their biocompatibility and clinical applicability, polymeric nanoparticles offer greater flexibility in controlled release and surface functionalisation. In contrast, inorganic nanoparticles exhibit intrinsic antimicrobial activity but are often limited by concerns about toxicity and long-term biocompatibility. Understanding these differences is essential for selecting the appropriate platform based on the specific therapeutic objective and disease context.

#### 2.1.1. Lipid-Based Nanocarriers

Liposomes are spherical vesicles that are made up of one or more phospholipid bilayers that enclose an aqueous core, with the size of the liposomes usually varying between 50 and 500 nm. Their structural flexibility allows hydrophilic drugs to be encapsulated in the aqueous core, lipophilic drugs to be incorporated into the lipid bilayer, and amphiphilic molecules to be incorporated at the bilayer interface [37]. Liposomes exhibit high biocompatibility, with their composition able to resemble natural cell membranes and be modified to fuse with target cells for intracellular delivery. PEGylation, a surface modification with polyethene glycol (PEG), prolongs circulation half-life by decreasing opsonisation and the ensuing mononuclear phagocyte system (MPS) clearance [38]. In respiratory use, liposomes between 50 and 150 nm in diameter are the most efficient at balancing alveolar uptake (favoured by particles > 200 nm) and aerosolisation efficiency [39].

An example of the clinical translation of liposomal formulations in respiratory infection is liposomal amikacin inhalation (Arikayce^®^), which was approved by the FDA in 2018 and is being used to treat refractory Mycobacterium avium complex (MAC) lung disease [34]. This formulation contains the aminoglycoside antibiotic amikacin as neutral liposomes (diameter of liposomes is approximately 300 nm), enabling once-daily delivery of the aminoglycoside, amikacin, via nebulisation, which produces high and sustained drug concentrations in alveolar macrophages—the major intracellular niche of MAC [40]. In Phase 3 trials, Arikayce^®^ significantly improved sputum culture conversion rates compared to guideline-based therapy alone (29% vs. 9%), demonstrating the feasibility and clinical value of pulmonary nanomedicine delivery [41].

Recent advancements have expanded the liposome platform. Ligand-targeted liposomes combined with phenylboronic acid achieved targeted binding to inflamed pulmonary tissue via boronate-cyclodextrin coordination with sialic acid, thereby increasing pulmonary drug distribution and preserving the intestinal microbiota environment [42]. Lipid nanoparticles (LNPs) enable efficient pulmonary delivery of siRNA, protecting it from degradation and facilitating intracellular release for gene silencing. They show rapid uptake in activated immune cells and induce significant anti-inflammatory effects by suppressing TNF-α and related cytokines in lung injury models [43].

Polymer-based siRNA delivery systems have demonstrated efficient gene silencing in the lungs, achieving approximately 70% knockdown of TNF-α in murine models of inflammation. pH-responsive liposomes are engineered to destabilise and release their payload under acidic conditions, particularly within endosomal compartments (pH ~5.0–6.5) and can also respond to the mildly acidic microenvironment of inflamed or infected tissues.

The second generation of lipid-based systems includes solid lipid nanoparticles (SLNs) and nanostructured lipid carriers (NLCs), which exhibit better stability and release profiles than traditional liposomes. SLNs are composed of a solid lipid backbone (e.g., triglycerides, fatty acids, and waxes), which are solid at body temperature, whereas NLCs use liquid lipids to form an imperfect matrix with a greater drug loading capacity and less expulsion of the drug during storage [44]. Orally administered solid lipid nanoparticles (SLNs) loaded with antitubercular drugs (rifampicin, isoniazid and pyrazinamide) maintained therapeutic drug levels in plasma for up to 8 days and in organs for up to 10 days in murine tuberculosis models, enabling reduced dosing frequency compared to daily oral administration [45]. NLCs that entrap ciprofloxacin demonstrated greater biofilm penetration and killing of *P. aeruginosa* in CF sputum in comparison to the free drug [46].

Despite their lipid-based nature, nanocarriers are not without limitations. Issues such as drug leakage, physical instability during storage, and relatively high production costs may limit their widespread application. Additionally, although their clinical translation is more advanced than that of other systems, their performance can be affected by formulation-specific factors, including lipid composition and particle-size distribution.

The pulmonary route is one of the most clinically translatable routes of administration for nanomedicine-based therapy, allowing for direct delivery to the site of infection with minimal systemic uptake and resulting toxicity. Inhalation platforms, in particular nebulisers, can aerosolise liposomes, polymeric nanoparticles and lipid nanoparticles without the need for extensive powder engineering. Nebulized formulation is particularly suitable for critically ill patients and patients with impaired inspiratory capacity. However, residual drug loss and variability in aerosol output remain important limitations [25].

In contrast, dry powder inhalers offer greater formulation stability and ease of use for patients and portability. Nanoparticles are usually added to the microparticle formulation by spray drying or spray freeze-drying to achieve an aerodynamic diameter of about 1–5 μm, which is suitable for deep-lung deposition. Nevertheless, challenges in formulation to ensure the integrity of the nanoparticles during powder manufacture, to enable rapid redispersal after deposition, and to prevent aggregation in the presence of moisture remain significant [47].

Metered-dose inhalers (MDIs) allow for rapid and reproducible delivery of the drug but are not as well suited for nanoparticle formulation because of the difficulty in maintaining suspension stability, propellant compatibility, and dose uniformity. The preclinical results are encouraging, but if inhaled nanomedicines are to be successfully translated into the clinic, it is necessary to optimize aerosol performance, the aerodynamic properties of the particles, storage stability, sterilization methods, manufacturing methods on a large scale, and reproducible dose delivery, whilst maintaining the physicochemical characteristics of the nanoparticles during aerosolization [48,49].

#### 2.1.2. Polymeric Nanoparticles

Polymeric nanoparticles offer exceptional versatility through tunable degradation kinetics, surface functionality, and drug release profiles [50]. They are typically prepared from biodegradable, biocompatible polymers and have diameters ranging from 50 to 300 nm. However, the clinical translation of polymeric nanoparticles remains constrained by several challenges, including potential toxicity of degradation products, variability in manufacturing processes, and regulatory complexities. While their tunable properties offer significant advantages, ensuring reproducibility and safety at scale remains a key hurdle for their clinical implementation.

Synthetic polymers, especially polylactic-co-glycolic acid (PLGA), have been of significant interest because they can be used in a variety of therapeutic applications, have well-characterised degradation kinetics (hydrolysis to lactic and glycolic acids, which are metabolised by the citric acid cycle), and can protect a wide variety of antibiotics with high efficiency [51]. In the case of respiratory infection, 14-day sustained drug release after a single intratracheal injection of PLGA nanoparticles encapsulating azithromycin in murine pneumonia models was much higher than that with the freely dispersed drug (azithromycin) administered daily [52]. The ability to reduce dosing frequency to once a week is a significant benefit for chronic infections that require long-term treatment.

pH-responsive polymeric nanoparticles exploit the mildly acidic microenvironment of infected lung tissue (pH ~5.5–6.5) to enable targeted drug delivery. Charge-reversal polymer-functionalised nanoparticles, such as DA-AZI NPs, undergo surface charge switching under acidic conditions, exposing a positively charged core that enhances electrostatic interactions with negatively charged bacterial membranes. This facilitates deep biofilm penetration and improved local antibiotic delivery, with studies showing near-complete biofilm penetration within 40 min [53].

Natural polymers, such as chitosan, alginate, gelatin, and hyaluronic acid, offer biocompatibility, mucoadhesive properties (which may be useful for long-term residence in the airways), and, in certain cases, inherent antimicrobial activity [54,55]. A well-liked approach to pulmonary delivery is the use of chitosan, a cationic polysaccharide derived from chitin, which can temporarily open tight junctions between epithelial cells, thereby increasing paracellular delivery of the encapsulated drug [56]. Another direct antimicrobial activity of chitosan nanoparticles is based on the same principle: the electrostatic interaction of the nanoparticles with the negatively charged bacterial membranes disrupts the membrane and leads to the leakage of intracellular compounds [57].

Natural cell membrane-coated biomimetic nanoparticles possess the targeting properties of biological membranes and the capacity to deliver drugs, similar to synthetic cores. Macrophage membrane-coated nanoparticles (MNPs) preserve surface receptors and homing capacity of source macrophages, allowing them to be actively targeted to inflamed lung tissue and infected macrophages after systemic delivery [58]. MNPs containing an antimicrobial peptide lowered bacterial load and significantly minimised the production of cytokines of the inflammatory response in a murine model of *P. aeruginosa* pneumonia compared to free peptide and uncoated nanoparticles [59].

Among the new generation of polymeric nanocarriers are conducting polymers (CPs) like polypyrrole, polyaniline (PANI), and poly(3,4-ethylenedioxythiophene) (PEDOT) that possess unique properties of electrical conductivity and stimuli-responsiveness. Hybrid systems with natural polymers such as chitosan, alginate or gelatin are synthesized using the combination of biocompatibility and biodegradability of natural polymers and tunable physicochemical properties of synthetic CPs. The multifunctional nanocarriers have shown superior drug loading, stimuli-responsive and controlled drug release, cellular uptake and surface functionalization for targeted delivery. Their application in respiratory antimicrobial therapy remains in its infancy, but additional improvements in biocompatibility, biodegradability and long-term safety would be conducive to future clinical translation [60,61].

CP-based nanocarriers have different mechanisms compared to the natural biopolymer- and peptide-based nanocarriers. The main advantage of using natural biopolymers, such as chitosan and alginate, is that they have the ability to increase the antimicrobial activity in several ways, including increased biocompatibility and mucoadhesion and by allowing controlled drug release. In contrast, peptide-based nanocarriers can deliver targeted delivery by selective interactions with bacterial membranes. CPs, such as polypyrrole (PPy), polyaniline and PEDOT, have electrical conductivity and stimuli-responsive properties enabling an external trigger for drug release and surface functionalization. Additionally, the bactericidal property of CPs is affected by their oxidation (doping) status and polymer chain structure, which affect the surface charge density, hydrophobicity and interactions with the bacterial cell membranes. For example, proton-doped polyaniline exhibits higher antibacterial activity owing to enhanced electrostatic interactions with the negative surface charge of bacteria. Optimized polymer architecture also has been found to create PEDOT-based composites with enhanced antimicrobial properties and controlled therapeutic release [60,61,62,63].

Despite these advantages, synthetic conductive polymers also present important limitations that currently restrict their biomedical translation. Most conductive polymers are water-insoluble, with most notably being PANI, which needs modification or blending with hydrophilic polymers to enhance their processability and biological compatibility [64]. In addition, electrical conductivity is generally achieved through protonic or oxidative doping, often using strong acids, which may raise concerns regarding residual dopants, long-term stability, and biocompatibility. Furthermore, the cytotoxicity of conductive polymers is influenced by their chemical composition, dopants, degradation products, and surface characteristics, highlighting the need for careful material design and comprehensive safety evaluation before clinical application [65,66].

#### 2.1.3. Inorganic Nanoparticles

Metal nanoparticles such as silver (AgNPs), gold (AuNPs), zinc oxide (ZnO NPs), and copper oxide (CuO NPs) exhibit inherent antimicrobial activity through mechanisms fundamentally different from those of traditional antibiotics [67].

In addition to conventional metallic nanoparticles, metalloid nanomaterials such as tellurium nanoparticles (TeNPs) have also demonstrated promising antimicrobial activity. For example, chitosan-coated tellurium nanoparticles exhibited potent activity against multidrug-resistant Gram-positive bacteria, including methicillin-resistant Staphylococcus aureus (MRSA), with minimum inhibitory concentrations of 4–8 μg/mL while maintaining low cytotoxicity toward mammalian cells, highlighting their potential as biocompatible inorganic antimicrobial nanomaterials [57].

Silver nanoparticles are the best researched and are effective against a wide spectrum of pathogens, including MDR strains such as MRSA and *P. aeruginosa* [68]. AgNPs can exert their antimicrobial effects through the following mechanisms: (i) release of Ag+ ions that disrupt electron transport chains, disrupt ATP synthesis, and generate reactive oxygen species (ROS); (ii) direct membrane damage by interaction of the nanoparticle with the bacterial surface; (iii) DNA damage via ROS-mediated and direct binding; and (iv) inhibition of ribosome function and protein Interestingly, plant extract–AgNPs synthesised with a green method (e.g., *Phoenix dactylifera*, *Azadirachta indica*) have better antimicrobial activity and lower cytotoxicity than the chemically synthesised ones, which is explained by the surface coverage with phytochemicals that stabilise nanoparticles and additive antimicrobial properties [69].

The advantages of gold nanoparticles (AuNPs) include their easy functionalisation with targeting ligands (e.g., antibodies, antimicrobial peptides, aptamers), their high biocompatibility, and their use for photothermally killing bacteria [70,71]. Antimicrobial-conjugated AuNPs (e.g., buforin II, magainin I) can penetrate MRSA biofilms and kill both planktonic and biofilm-associated bacteria without triggering the development of resistance, since the physical membrane disruption process is not susceptible to conventional resistance mutations [72].

Similarly, zinc oxide nanoparticles (ZnO NPs) exhibit antimicrobial activity through multiple complementary mechanisms. In addition to photocatalytic generation of reactive oxygen species (ROS), which is enhanced under ultraviolet or visible-light irradiation, ZnO nanoparticles also demonstrate light-independent antibacterial activity through Zn^2+^ ion release, disruption of bacterial membrane integrity, and induction of oxidative stress under physiological conditions [73,74]. Consequently, although irradiation can enhance their antimicrobial efficacy, ZnO nanoparticles retain antibacterial activity in the absence of external light, supporting their potential for biomedical applications while emphasizing the need for optimized nanoparticle design and biocompatibility for in vivo use [75].

Zinc oxide nanoparticles (ZnO NPs) exhibit broad-spectrum activity against Gram-positive and Gram-negative bacteria, destabilising membranes, releasing Zn^2+^ ions, and causing oxidative stress [76]. ZnO NPs have a particularly bright future in pulmonary applications, as they exhibit relatively low mammalian cytotoxicity compared with silver, intracellular activity against *M. tuberculosis* in macrophages, and the ability to tune host immune responses [77]. The expression of virulence factors in *P. aeruginosa* (protease, pyocyanin, and pyoverdine) has been demonstrated to be inhibited by sub-inhibitory concentrations of ZnO NPs via interference with quorum-sensing pathways, lowering pathogenicity without subjecting the pathogen to strong selective pressure to resist [78].

Quantum dots (QDs) and carbon-based nanomaterials, such as graphene oxide (GO) and carbon nanotubes (CNTs), produce light-induced ROS to achieve photodynamic antimicrobial therapy. QDs have been explored due to their capacity to generate singlet oxygen when exposed to visible light, killing planktonic and biofilm-dwelling bacteria through oxidative damage to lipids, proteins, and DNA. Pulmonary application challenges include the potential for cytotoxicity, low biodegradability, and limited light delivery to deep lung areas, which is technically difficult [79].

Recent progress in biomineralized inorganic nanocarriers further demonstrates that rational materials engineering can enhance carrier biocompatibility, payload stability, and intracellular delivery, thereby expanding the therapeutic potential of multifunctional inorganic nanoplatforms [80].

Despite their potent antimicrobial properties, the clinical application of inorganic nanoparticles is limited by concerns about cytotoxicity, tissue accumulation, and limited biodegradability. These safety considerations remain a major barrier to their translation into clinical practice, particularly for pulmonary applications where long-term exposure may pose additional risks.

Collectively, these nanocarrier systems offer distinct advantages and limitations, highlighting that no single platform is universally optimal for all respiratory infections. Instead, the selection of an appropriate nanocarrier should be guided by the specific biological barrier being targeted, the drug’s pharmacokinetic requirements, and the clinical context. This underscores the importance of a rational, application-driven approach in the design of nanotechnology-based therapies.

Although the antimicrobial activity of these inorganic nanoparticles is quite high, their clinical use is restricted due to their cytotoxicity, tissue accumulation, lack of biodegradability, and high oxidative stress because of their prolonged exposure. Although their safety profile has been enhanced in recent years through advances in nanoparticle surface engineering and biocompatible formulations, there is still a need for comprehensive long-term studies to evaluate their pulmonary biodistribution, immunogenicity and repeated-dose toxicity prior to their widespread clinical translation, especially for inhaled formulations [81]. Therefore, the optimization of the composition, dose, and functionalization of the surface of nanoparticles is crucial to achieve the desired antimicrobial effects at the highest dose possible with the least amount of adverse biological effects. Further research is needed to conduct detailed long-term safety studies of biodistribution, clearance, biodegradation, and repeated-dose pulmonary toxicity to enable the safe clinical implementation of inorganic nanomaterials for pulmonary infections [82]. An overview of the major inorganic nanoparticles used for respiratory drug delivery is presented in Table 1.

To overcome these limitations, nanomaterials with more biocompatibility have been at the centre of future studies, including naturally derived polymeric nanoparticles, lipid-based nanocarriers and green-synthesised metallic nanoparticles. Compared to conventionally synthesized inorganic nanoparticles, these materials exhibit better biodegradability, biocompatibility, low immunogenicity and low systemic toxicity. In addition, the green synthesis process by using plant extracts or microbial biomolecules eliminates the use of toxic chemical-reducing agents and improves bifunctionality on the surfaces of nanoparticles. These properties make the platforms potential candidates for repeated administration and prolonged use of antimicrobials [83].

Nanocarriers have great therapeutic potential, but there are still limitations about their long-term toxicity that hinder their clinical translation. Particles inhaled into the lungs can cause oxidative stress, inflammation, damage to the lungs’ epithelium, and disruptions to immune homeostasis. Particle composition, size, surface chemistry, administered dose and exposure duration are the main factors determining the extent of toxicity. While surface functionalization may reduce immunogenicity and nonspecific protein adhesion, it may also result in complement activation and anti-polymer immune responses and in accelerated clearance, all of which can lead to decreased therapeutic efficacy with repeated administration. Following inhalation, nanoparticles have multiple routes of clearance, including mucociliary transport, phagocytosis by alveolar macrophages, lymphatic drainage, epithelial uptake and translocation to systemic circulation. All these mechanisms impact on the pulmonary retention and the biodistribution. Thus, it is essential to thoroughly evaluate biodistribution and biodegradability, clearance kinetics, immunogenicity, and repeated-dose pulmonary toxicity before widespread clinical use of nanomedicine-based antimicrobial therapies [84,85].

**Table 1:** Overview of inorganic nanoparticles for respiratory drug delivery.

Nanoparticle Type	Typical Size Range	Key Physiological Properties	Primary Antimicrobial Mechanism	Relevance to Respiratory AMR	Reference
Silver (AgNPs)	10–100 nm	High surface area, ROS generation, Ag^+^ release	Membrane disruption, ROS, DNA damage, protein inactivation	Strong activity against MDR pathogens and biofilms in respiratory infections	[67,68]
Gold (AuNPs)	5–150 nm	Easily functionalised, biocompatible, and the SPR effect	Photothermal heating, ROS (with NIR), membrane binding	Enables targeted biofilm disruption and controlled therapy via external triggers	[70,71]
Zinc oxide (ZnO)	20–200 nm	High stability, Zn^2+^ release, photocatalytic	ROS generation, membrane destabilisation, Zn^2+^ toxicity, quorum-sensing inhibition	Low mammalian toxicity, anti-biofilm, and immune modulation	[76,77]
Copper oxide (CuO)	20–100 nm	Redox activity, Cu^2+^ release	ROS, protein oxidation, membrane damage	Broad-spectrum antibacterial activity; effective in disrupting bacterial membranes and metabolic processes	[86]
Mesoporous silica nanoparticles (MSNs)	50–300 nm	High pore volume, tunable pore size, surface functionalisation	Carrier for antibiotics; limited intrinsic activity	High drug loading, controlled release, biocompatible	[87,88]
Quantum dots (QDs)	2–20 nm	Size-tunable fluorescence, photoexcitation	ROS, membrane damage	Imaging and therapy (theranostic)	[79]

ROS, Reactive oxygen species; NIR, Near-infrared; SPR, Surface plasmon resonance.

### 2.2. Mechanisms: How Nanotechnology Helps in Combating AMR

**Figure 1:**
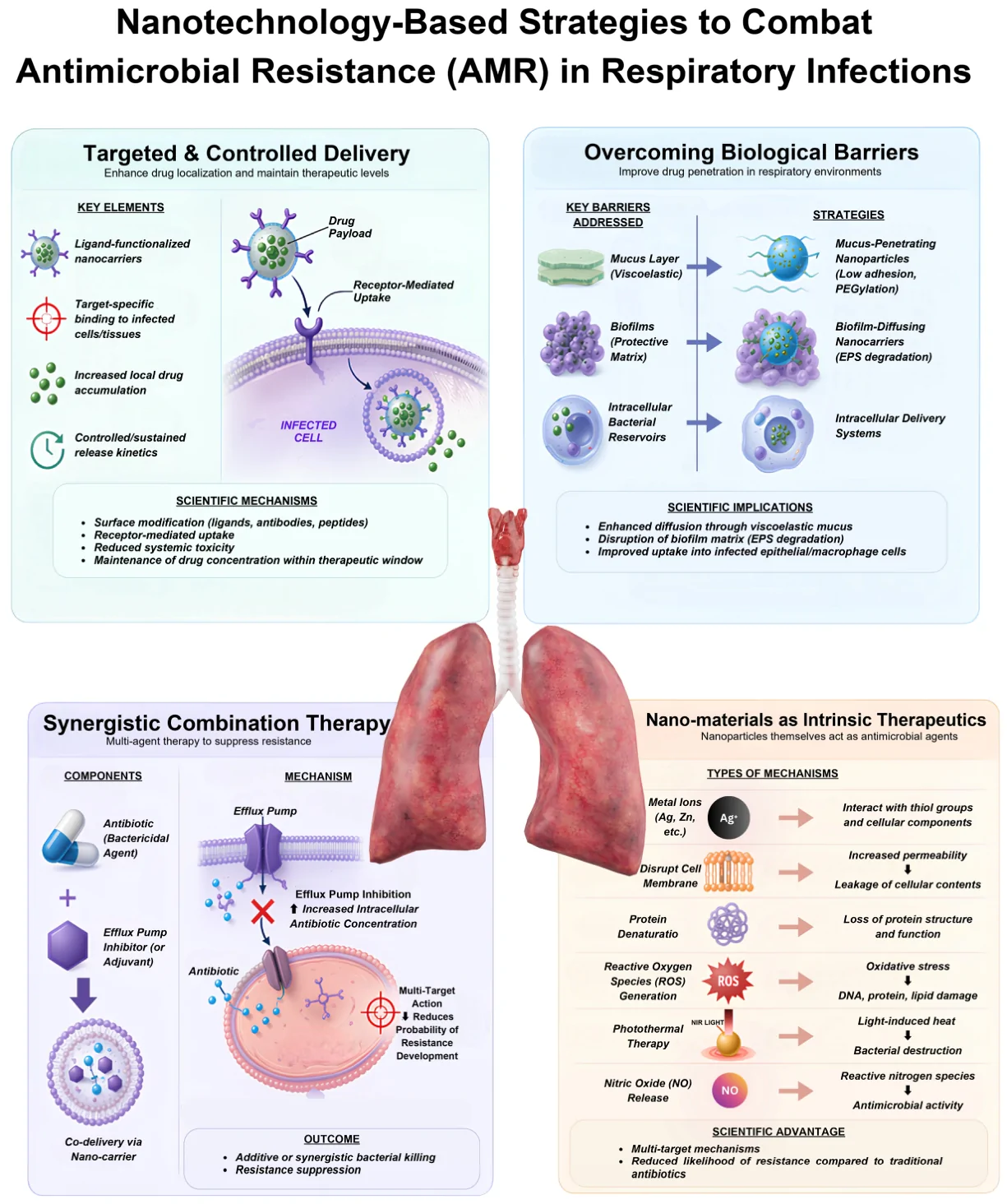
Schematic representation of nanotechnology-based mechanisms to combat antimicrobial resistance in respiratory infections. ↑ indicates increased intracellular antibiotic concentration; ↓ indicates reduced probability of resistance development.

This core section details the specific strategies by which nanocarriers overcome the resistance mechanisms and biological barriers that compromise the efficacy of conventional antibiotics in respiratory infections (Figure 1). Broadly, nanotechnology-based strategies for combating antimicrobial resistance can be categorised into four principal mechanisms: (1) enhanced penetration across biological barriers such as mucus and biofilms; (2) targeted delivery to infected cells or pathogens; (3) controlled and sustained drug release to maintain effective therapeutic concentrations; and (4) the introduction of alternative antimicrobial mechanisms independent of conventional antibiotics. This framework provides a structured basis for understanding how nanocarriers improve therapeutic outcomes in respiratory infections.

#### 2.2.1. Targeted and Controlled Delivery

Active targeting is a major strength of nanocarriers compared to traditional antibiotics, enabling selective accumulation at sites of infection with minimal off-target accumulation [89]. However, it is important to note that the effectiveness of active targeting strategies may be influenced by factors such as receptor heterogeneity, dynamic changes in the inflammatory microenvironment, and limited penetration into dense biofilm structures. As a result, the benefits observed in controlled experimental settings may not always translate directly into clinical outcomes. The carrier can be actively directed to receptors or surface structures expressed on specific cells or bacteria that are the pathogens or infected cells by functionalising the surface of the nanoparticles with a particular ligand, e.g., antibodies, antibody fragments (Fab, scFv), peptides, aptamers, or carbohydrates [90].

In the case of respiratory infections, targeting has taken advantage of a number of unique properties of the infected lung microenvironment: Lectin-based targeting relies on carbohydrate-binding proteins (lectins) present on bacterial surfaces and infected host cells. Mannose-functionalised nanocarriers recognise the mannose receptor (CD206), which is very abundant on alveolar macrophages and dendritic cells—the main host cells of the *M. tuberculosis* and other intracellular pathogens [91]. Mannosylated liposomes that carry rifampicin were 5-fold more taken up by infected macrophages and 3-fold more intracellularly killed *M. tuberculosis* than non-targeted liposomes [92]. While these findings are promising, most evidence remains limited to preclinical models, and further clinical validation is required to determine their real-world applicability.

Peptide-mediated targeting uses short peptide sequences, either discovered by phage display or rationally designed, that bind to bacterial surface structures. The antimicrobial peptide ubiquicidin (UBI) and its analogues selectively bind to bacterial membranes via electrostatic interactions, facilitating selective delivery to the site of infection [93]. Nanoparticles of UBI-conjugated PLGA with clarithromycin demonstrated mean inhibition zone diameters against *Salmonella typhi*, *Haemophilus influenzae*, and *Escherichia coli* that were significantly higher [94].

Despite recent progress, intracellular drug delivery is still a challenge for the treatment of *Mycobacterium tuberculosis* (Mtb). The pathogen can survive within alveolar macrophages by disrupting phagosomal maturation, escaping lysosomal degradation, and remaining metabolically inert, with decreased susceptibility to antibiotics [95]. Traditional antibiotics are often not retained well within infected macrophages, do not accumulate well in the cytosol, and have poor penetration into granulomatous lesions. These restrictions require long-term multiple drug therapy, which has a potential risk of toxicity, low compliance and development of multiple drug-resistant strains [96]. To overcome these obstacles, nanocarrier-based drug delivery systems have been engineered with macrophage-targeting peptides, such as mannose, to promote controlled intracellular drug release and endosomal escape, thereby increasing antibiotic availability in the cytosol [97]. However, there are a number of barriers, such as variability in granuloma structure, limited access into the necrotic lesions, differing macrophage phenotypes, and the need for concurrent delivery of sufficient and safe levels of drug to the cells while minimising long-term toxicity. Optimisation of the intracellular targeting strategies and testing in clinically relevant models of tuberculosis is needed before widespread clinical translation [98].

Nanocarriers based on aptamers use short single-stranded DNA or RNA oligonucleotides selected by systematic evolution of ligands by exponential enrichment (SELEX) to bind target molecules with high affinity and specificity [99]. Aptamers offer advantages over antibodies, including lower immunogenicity, smaller size, and improved stability. Aptamer-functionalised nanoparticles have been explored for targeted delivery to *P. aeruginosa*, and outer membrane proteins such as OprF represent promising targets for species-specific binding. However, the integration of antibiotic-loaded mesoporous silica nanoparticles for selective killing in mixed bacterial cultures is still under investigation [100,101].

Antibody-functionalised nanocarriers exploit the high specificity of the monoclonal antibodies on bacterial surface antigens. Although this strategy remains in its early stages for infectious diseases, nanocarriers functionalised with antibodies targeting *P. aeruginosa* type IV pili or S. aureus protein A have demonstrated enhanced bacterial targeting and therapeutic efficacy in preclinical models [101,102]. Controlled release adjusts the pharmacokinetics of encapsulated drugs to achieve therapeutic levels at the site of infection over prolonged periods, reducing the frequency of dosing, minimising toxicity during the peak phase, and possibly inhibiting the development of resistance by preventing low therapeutic trough levels [103]. Encapsulation of nanoparticles can convert quickly excreted antibiotics (with half-lives of minutes to hours) into sustained-release preparations that maintain therapeutic concentrations for days to weeks.

Recent advances in biocompatible nanoplatform engineering have further improved intracellular and cytosolic delivery through rational carrier design, highlighting the potential of multifunctional delivery systems to enhance therapeutic efficiency while minimizing systemic toxicity [81].

Release kinetics can be engineered to achieve distinct therapeutic outcomes. A zero-order release system provides a constant, steady release of the drug over time, avoiding fluctuations in drug concentration and ensuring consistent antimicrobial activity. Such profiles are typically achieved through diffusion-controlled polymer matrices or the gradual erosion of biodegradable carriers, as demonstrated by PLGA nanoparticles that deliver levofloxacin with sustained release for up to 120 h in lung fluid [104,105]. In contrast, biphasic release systems have an initial burst release to quickly attain therapeutic concentrations, followed by a sustained phase to maintain drug exposure, a design especially beneficial in acute and severe infections. Unlike solitary-phase release systems, chitosan-coated tobramycin-loaded liposomes exhibit an initial release stage followed by sustained delivery, resulting in a higher rate of *Pseudomonas aeruginosa* biofilm eradication than single-phase release systems [106].

More advanced methods include stimulus-responsive release systems, in which infection-specific stimuli can spatiotemporally regulate drug release. The pH-responsive systems among the endogenous triggers are based on the fact that the microenvironment of infected tissues is slightly acidic, and the protonation of the polymer components causes immediate drug release. In contrast, the system remains stable at the physiological pH of 7. Pathogen-derived enzymes can act as endogenous triggers for drug release, providing a highly selective strategy for combating antibiotic-resistant respiratory infections. Rapid degradation of carrier matrices and subsequent release of encapsulated antibiotics in the presence of resistant bacteria have been demonstrated with β-lactamase-responsive systems. For example, a cephalosporin-crosslinked hydrogel degraded upon exposure to β-lactamase-producing *P. aeruginosa*, triggering the release of ciprofloxacin-loaded liposomes [107,108].

Moreover, toxin-responsive systems also exploit bacterial virulence factors, like pore formation caused by toxins such as α-toxin of *Staphylococcus aureus*, which, when incorporated into the liposomal membrane, creates nanoscale pores, resulting in rapid diffusion of encapsulated antibiotics, such as vancomycin, with 48% of drug release occurred in 30 min in the presence of MRSA, leading to effective bacterial growth inhibition [109].

Exogenous triggers: An externally triggered system offers precise spatiotemporal control over drug release. For example, magnetically controlled nanocarriers incorporating a superparamagnetic iron oxide core generate localized heat under an alternating magnetic field (AMF), which induces cleavage of a thermally reversible Diels–Alder covalent linkage (typically formed between furan and maleimide functional groups). This retro-Diels–Alder reaction destabilizes the carrier matrix and enables controlled release of the encapsulated therapeutic payload [110]. Similarly, near-infrared (NIR) light-responsive nanocarriers provide precise, controlled drug release, where absorbed light is converted to heat or reactive species, which in turn trigger bond cleavage and drug release [111].

#### 2.2.2. Overcoming Biological Barriers

One of the transformative capabilities of nanotechnology is that it can circumvent the several biological obstacles that prevent traditional antibiotics from reaching, isolating, entrapping, or inactivating pathogens in the respiratory tract. These obstacles, including the airway surface liquid and mucus gel layer of the airways, bacterial biofilms and host cell membranes, are sequential barriers that an effective antimicrobial must overcome to achieve its goal [28]. To overcome each barrier, nanocarriers can be rationally designed with specific physicochemical characteristics to provide a degree of control not achievable with free drugs.

##### Size-Dependent Penetration

Nanoparticles are small, which enhances their penetrability through epithelial linings and into infected tissues. Intranasally administered PLGA nanoparticles spread widely throughout the lungs and are predominantly deposited in alveolar macrophages, the main host cells in respiratory infections such as *Mycobacterium tuberculosis.* Compared with uninfected cells, infected macrophages ingest much more nanoparticles, leading to higher drug levels within the cell and improved targeting of the infectious agent. Such preferential uptake was observed regardless of the model of bacterial, viral, or parasitic infection and across a variety of nanomaterials, implying a general property of nanocarrier systems [112].

##### Biofilm Penetration

Surface alterations enable nanoparticles to penetrate the thick extracellular polymeric matrix of biofilms. It has been shown that polyelectrolyte surfactant nanoparticles can be targeted to overcome thickened mucus and biofilm formation, which inhibit the penetration of conventional cationic antimicrobials such as tobramycin and polymyxin B in patients with *P. aeruginosa* infections of the cystic fibrosis [113]. The production of these nanoparticles is based on graft copolymers composed of polyetheramine side chains and an anionic poly(alkyl acrylic acid) backbone, which forms electrostatic interactions with cationic antimicrobials and self-assembles. Tuning backbone chemistry and graft density allows the control of particle size, drug binding strength and release kinetics [114,115].

##### Biofilm Disruption

In addition to facilitating penetration through the extracellular polymeric substance (EPS), nanomaterials actively disrupt the structural integrity of established biofilms through several complementary mechanisms. Cationic nanoparticles interact electrostatically with negatively charged EPS components, weakening the biofilm matrix and increasing its permeability. Nanocarriers can also co-deliver antibiotics with matrix-degrading enzymes, such as DNase I or dispersin B, which degrade extracellular DNA and polysaccharides that maintain biofilm stability. Furthermore, several nanomaterials interfere with quorum-sensing pathways responsible for biofilm maturation and maintenance, thereby reducing extracellular polymer production and promoting biofilm dispersal. Metallic nanoparticles, including silver and zinc oxide nanoparticles, further enhance biofilm eradication through the generation of reactive oxygen species, membrane disruption, and metabolic inhibition, thereby rendering biofilm-associated bacteria more susceptible to antimicrobial therapy.

Modulation of the interaction between nanocarriers and extracellular polymeric substances (EPSs) has been a major improvement in recent years, enhancing their effectiveness in penetrating bacterial biofilms. Hydrophilic polymer modifications like polyethylene glycol (PEG) or zwitterionic coatings help prevent non-specific binding to mucins and EPS and facilitate diffusion through dense biofilm matrices [116]. In the mildly acidic environment of the biofilm, charge-switchable nanoparticles become positively charged, increasing electrostatic interactions with bacterial membranes and leading to deeper penetration [117]. Targeted nanocarriers containing antimicrobial peptides, antibodies, lectins, or other anti-biofilm agents promote selective uptake at infection sites and promote efficient uptake of bacteria [118]. Site-specific and on-demand drug delivery is achieved by stimuli-responsive nanocarriers, which release drugs upon infection-associated stimuli, such as acidic pH, bacterial enzymes, reactive oxygen species and toxins. These systems deliver the highest local antibiotic levels without untimely leakage and possible systemic toxicity [116]. Multifunctional nanoplatforms with enzyme-mediated EPS degradation, quorum-sensing inhibition and controlled antibiotic release, combined with photothermal or photodynamic therapy, provide a more complete disruption of the mature biofilms, better penetration into persister-cell-rich areas and higher bacterial eradication rates than conventional antibiotic formulations [119,120].

Neutrophil membrane-encapsulated nanosonosensitizers (Fe/TNT@NM) have been developed using a biomimetic approach to the treatment of *P. aeruginosa* pneumonia. This system produces extracellular sonodynamic reactive oxygen species upon ultrasound irradiation and increases the release of Fe^3+^. The extracellular Fenton reaction catalysed by these ions enhances chemodynamic responses and interferes with intracellular iron homeostasis, leading to ferroptosis in bacteria. The neutrophil membrane coating promotes immune evasion and biofilm-targeted delivery. Fe/TNT@NM inhibits biofilm formation and prevents lung damage in a murine pneumonia model [121].

The delivery of antimicrobial peptides (AMPs) using nanoparticles is another approach that could help overcome biological barriers to treating multidrug-resistant ventilator-associated pneumonia (VAP). VAP, which is mainly caused by Gram-negative bacilli that are resistant to multiple drugs, is a significant problem in intensive care units. AMPs are promising therapeutic agents because they are essential elements of the innate immune system and have broad-spectrum activity, but their enzymatic breakdown, low bioavailability, short systemic half-life, and potential cytotoxicity limit their clinical use. To overcome these barriers, nanotechnology-based delivery systems that can be used to deliver AMPs include liposomes, dendrimers, and polymeric nanoparticles, which have been used to protect AMPs against enzymatic degradation, improve solubility, target delivery to the lungs, and minimise side effects on non-target tissues. Infection-specific stimulus-responsive nanoplatforms that produce AMPs in response to a specific disease state, e.g., acidic pH or enzymatic activity, are even more precise in treatment and minimise systemic exposure. Nanoparticle delivery of AMP has shown clinical potential in preclinical studies, demonstrating its effectiveness against multidrug-resistant VAP [122].

Man-mPDA NPs are developed as a photothermal agent and a rifampicin delivery vector using mesoporous polydopamine nanoparticles (mPDA NPs) as the target to macrophages. These nanoparticles exhibited specific macrophage-targeting effects. Photothermal therapy using Rif@Man-mPDA NPs might provide a synergistic effect by targeting drug delivery to mediate better intracellular clearance of *M. tuberculosis* through autophagy-stimulated macrophage activation by host immune responses. This approach was successful in a mouse model of cutaneous tuberculosis, inhibiting Mtb burden and relieving pathological lesions, with minimal systemic side effects [123].

#### 2.2.3. Synergistic Combination Therapy

Nanoparticles provide a platform for co-delivery, enabling a single carrier to deliver two or more therapeutic agents to achieve synergistic activity. This strategy increases efficacy against resistant strains and can help prevent the development of resistance by targeting bacteria in various ways.

##### Silver Nanoparticles

An overview of the literature, as presented by Casals et al., demonstrates that controlled release of silver ions by silver nanoparticles (AgNPs) enhances the effects of traditional antibiotics. The silver response by the bacterium is energetically demanding, weakening bacterial metabolism and, consequently, overwhelming bacterial defence, thereby enhancing the effect of the antibiotics [124]. AgNPs have been extensively utilised to augment the efficacy of current antibiotics against various MDR pathogens [125].

##### Quorum-Sensing Inhibitors (QSIs) Combined with Antibiotics

QSIs disrupt bacterial cell–cell communication and biofilm formation, thereby enhancing the antibiotics’ efficacy. Ho et al. have invented self-assembling squalenyl hydrogen sulfate nanoparticles (SqNPs) that could be used to deliver tobramycin and a novel lipophilic QSI simultaneously. These nanocarriers were found to have very high loading capacities—30% of tobramycin and about 10% for QSI—and improved biofilm penetration resulting in total elimination of *P. aeruginosa* biofilms at about 16-fold reduced tobramycin concentration in comparison with tobramycin alone [126].

##### Efflux Pump Inhibition

The application of nanocarrier-based drug delivery systems has emerged as one option to overcome efflux-mediated resistance and re-establish the efficacy of antibiotics. A study reports that metallic, polymeric, and lipid nanocarriers potentiate the antibiotic effect, lower minimum inhibitory concentrations (MICs), and decrease expression of bacterial efflux pumps [127]. Similarly, a review by Thillai et al. explains how nanobiotics can be engineered as nanoscale materials with antibacterial properties to inhibit efflux pump activity, increase drug delivery, and disrupt bacterial cell membranes to overcome traditional resistance mechanisms [128].

An optimised nanostructured lipid carrier (NLC) of Norfloxacin and an efflux pump inhibitor (2-amino-thiophen-6CN-Ethyl) showed high encapsulation efficiency (99.50% of the inhibitor and 90.91% of norfloxacin) and physicochemical stability up to 60 days. Antibacterial experiments revealed that the NLC formulation greatly enhanced the activity of norfloxacin against resistant *Staphylococcus aureus* strains that overexpress efflux pump genes, and the effect was better than that of the free drug combination [129].

##### Nanoparticle-Mediated Antimicrobial Peptide (AMP) Delivery

A review by Santhana Krishnan et al. discusses the use of nanotechnology to deliver AMPs to treat multidrug-resistant ventilator-associated pneumonia (VAP). Innate immune system essential elements, such as AMPs, have broad-spectrum activity and are therefore promising therapeutic agents; unfortunately, enzymatic degradation, low bioavailability, short systemic half-life, and possible cytotoxicity limit their clinical use. Nanotechnology-based delivery systems, such as liposomes, dendrimers, and polymeric nanoparticles, help prevent enzymatic degradation of AMPs, improve their solubility, deliver them directly to the lungs, and minimise side effects. The development of stimulus-responsive nanoplatforms, which discharge AMPs upon infection-specific signals such as acidic pH or enhanced enzymatic activity, also contributes to better treatment specificity and decreased systemic exposure [122].

##### Phage–Antibiotic Synergy (PAS) with Nanotechnology

Research has indicated that PAS is effective in the reduction in biofilm biomass of healthcare-associated infections. Examples of these include ampicillin/sulbactam in combination with phage SALSA, which eliminates Serratia marcescens SM01, and ciprofloxacin and phage vB_Eco4-M7, which reduce the biofilm density of Shiga toxin-positive *E. coli* O157:H7. Nanoscale encapsulation, especially the loading of phages into liposomes, promotes intracellular delivery, with superior membrane penetration, gastrointestinal survival, and intracellular delivery compared with free phages [130].

#### 2.2.4. Nanomaterials as Intrinsic Therapeutics

Beyond their use as passive carriers, some nanomaterials exert a direct antimicrobial effect through physicochemical mechanisms distinct from those of classical antibiotics. These innate activities provide alternative avenues to eliminate drug-resistant bacteria, which, in most cases, tend to attack multiple cell structures simultaneously, thereby reducing the likelihood of resistance developing.

##### Reactive Oxygen Species (ROS) Generation

Nanoparticles of metals and metal oxides catalyse the generation of ROS, such as superoxide, hydrogen peroxide and hydroxyl radicals. The antibacterial properties of silver nanoparticles (AgNPs) are mostly explained by their ability to generate ROS, which leads to oxidative stress on bacterial membranes, proteins, and DNA [131]. The mode of action of Zinc oxide (ZnO) nanostructures is similar to the ROS-mediated pathway [132]. Quantum dots and carbon-based nanomaterials (e.g., graphene oxide) produce light-induced ROS for photodynamic therapy, although challenges such as cytotoxicity and light delivery to deep lung regions remain [133].

##### Metal Ion Release

Dissolution of metallic ions—Ag^+^ (AgNPs), Zn^2+^ (ZnO NPs), Cu^2+^ (CuO NPs)—disrupts important bacterial processes. These ions disrupt electron transport chains, displace essential metal cofactors, and generate additional ROS via Fenton-type reactions. Due to multiple pathways of interest by metal ions, single mutations are unlikely to cause resistance [124,134].

##### Photothermal Therapy

Gold nanoparticles (AuNPs) and gold nanostars are effective in localised heating of bacteria by converting near-infrared (NIR) light into physical membrane disruption, leading to their death. An overview of research by Janani and ES indicates that the photothermal effect of AuNPs, which convert NIR light to heat, has demonstrated photothermal-based bacterial killing [135]. A recent study by Yao et al. reported an increase in the antibacterial activity of gold nanostars upon NIR irradiation, attributed to the combined effects of photothermal and photodynamic actions [136]. In addition, non-antibiotic copper sulfide/gold nanocluster nanoparticles (CuS/AuNCs@Lip NPs) were designed for the treatment of MRSA pneumonia. When excited at 1064 nm NIR, these nanoparticles demonstrated high levels of photothermal activity and ROS generation, rupturing bacteria membranes and biofilms. In vivo experiments demonstrated target enrichment at infection sites and high efficacy in MRSA pneumonia models, and biosafety was well-favoured [137].

##### Nitric Oxide (NO)-Releasing Nanoparticles

NO is a broad-spectrum antimicrobial cytokine produced endogenously by macrophages. It targets a variety of bacterial targets (DNA, iron-sulfur clusters, membrane lipids) and has not been shown to induce resistance. The gelatin nanoparticles (GNP/NO) that released NO were developed as an inhalable therapeutic. The aerodynamic diameter of the nebulised GNP was 4.0 ± 0.3 μm, which means that it was deposited in the lower respiratory tract. Antimicrobial testing against *S*. *aureus* and *P. aeruginosa* indicated total eradication of bacteria in bronchoalveolar lavage fluid, with no cytotoxicity to human pharyngeal cells [138].

#### 2.2.5. Integrating Nanocarrier Design with Pulmonary Barriers and Pathogen Biology

A central challenge in the application of nanotechnology to respiratory infections lies in aligning nanocarrier design with the complex biological barriers of the pulmonary environment and the pathogen-specific mechanisms of persistence. This challenge is magnified by the high and rising resistance rates among key respiratory pathogens; a recent network meta-analysis of over 365,000 clinical isolates found that carbapenems, the current last-line option, already face resistance rates of 17% against Enterobacteriaceae, 22% against *P. aeruginosa*, and 33% against *A. baumannii* [139]. While a wide range of nanocarrier systems and functional strategies have been described, their therapeutic success depends on the rational integration of physicochemical properties with disease-specific constraints.

At the airway level, the mucus barrier represents the first major obstacle to effective drug delivery. Dense, viscoelastic mucus limits the diffusion of conventional antibiotics and promotes drug sequestration. In this context, surface-engineered nanoparticles—particularly those with neutral or hydrophilic coatings such as polyethylene glycol (PEG)—demonstrate enhanced mucus penetration by reducing adhesive interactions with mucin glycoproteins [140]. However, this design must be carefully balanced, as reduced mucoadhesion may also limit retention time within the lung.

Biofilm-associated infections, particularly those involving *Pseudomonas aeruginosa*, introduce an additional layer of complexity. The extracellular polymeric matrix not only restricts antibiotic penetration but also creates a heterogeneous microenvironment characterised by metabolic dormancy and increased tolerance [17]. Nanocarriers designed for biofilm disruption typically incorporate charge-modulating or enzyme-responsive features, enabling deeper penetration and targeted release within the biofilm structure. Notably, systems that combine physical disruption mechanisms with antibiotic delivery appear more effective than monotherapy approaches.

Intracellular pathogens such as *Mycobacterium tuberculosis* require a fundamentally different delivery strategy. The intracellular localisation of these organisms within alveolar macrophages necessitates targeted uptake and controlled intracellular release [17]. Ligand-functionalised nanocarriers, particularly those exploiting macrophage-specific receptors (e.g., mannose receptor CD206), have demonstrated enhanced cellular internalisation and improved intracellular drug concentrations [141]. This highlights the importance of active targeting strategies in overcoming cellular-level barriers that are inaccessible to conventional antibiotics. Beyond physical barriers, enzymatic resistance (e.g., metallo-β-lactamases such as NDM-1) can also be tackled by adjuvant molecules; for instance, the repurposed drugs risedronate and methotrexate directly inhibit NDM-1 and restore carbapenem activity, an approach that could be combined with nanocarrier-mediated delivery [142].

Importantly, these biological challenges are not uniform across respiratory pathogens, and no single nanocarrier platform is universally optimal. Instead, the effectiveness of nanotechnology-based interventions depends on the extent to which carrier design is tailored to the dominant resistance mechanism—whether it is physical exclusion (mucus), structural protection (biofilms), or intracellular sequestration. This reinforces the concept that nanotechnology should be viewed primarily as a delivery-oriented solution, capable of enhancing drug accessibility and localisation rather than functioning solely as a novel antimicrobial modality. This tailored approach aligns with the broader understanding that AMR emerges from interconnected molecular, environmental, and clinical factors, as recently reviewed in the context of global public health [143].

Despite promising preclinical outcomes, the translation of these strategies into clinical practice remains limited. A small number of inhalable nanomedicine formulations, such as liposomal amikacin, have demonstrated clinical feasibility, yet most advanced systems remain at the experimental stage. This gap reflects not only technical and regulatory challenges but also the need for more disease-specific design frameworks that integrate pulmonary biology, pathogen characteristics, and pharmacokinetic requirements. Collectively, a rational, barrier-oriented approach to nanocarrier design provides a unifying framework for understanding how nanotechnology can be effectively applied to antibiotic-resistant respiratory infections. Future progress in this field will depend on moving beyond platform-driven development toward clinically informed, mechanism-based optimisation of nanomedicine strategies.

### 2.3. Spotlight on Key Respiratory Pathogens

The mechanisms by which nanotechnology overcomes antimicrobial resistance have been discussed in Section 2.2. This section focuses on applications of these strategies against major respiratory pathogens, emphasizing pathogen-specific therapeutic challenges and clinical outcomes.

#### 2.3.1. *Pseudomonas aeruginosa*

*P. aeruginosa* is the major cause of nosocomial pneumonia as well as chronic pulmonary infections in cystic fibrosis. Its capability to form biofilms and develop resistance mechanisms in an extremely short period of time renders it extremely challenging to eliminate with traditional antibiotics [126].

A biomimetic nanosonosensitizer (Fe/TNT@NM) composed of an iron-doped titanate nanotube core wrapped in a neutrophil membrane has been designed to combat biofilm-associated *P. aeruginosa* pneumonia. The neutrophil membrane coating facilitates biofilm-targeted delivery and immune evasion, while ultrasound activation enhances antibacterial activity through sonodynamic and chemodynamic effects, as described in Section 2.2. In a murine pneumonia model, Fe/TNT@NM significantly inhibited biofilm formation and alleviated pulmonary damage, demonstrating its potential for targeted treatment of persistent *P. aeruginosa* indection [121].

Moxifloxacin and the antibacterial peptide ε-poly-L-lysine were co-encapsulated in lipase-responsive nanoparticles with a weakly negative charge for treatment of multidrug-resistant *P. aeruginosa*. The formulation demonstrated excellent therapeutic efficacy by achieving approximately 99% bacterial clearance in lungs of infected mice while exhibiting minimal toxicity to pulmonary tissue [144].

The GEQ NPs, self-assembled from Ga^3+^, quercetin and ε-poly-L-lysine, represent multifunctional nanoplatform for treatment of *P. aeruginosa* biofilm associated infections. By simultaneously targeting multiple bacterial processes, the formulation exhibited potent antibiofilm activity and demonstrated the advantage of combining complementary antimicrobial strategies within a single-nanocarrier system [145].

Norharmane-loaded bacterial cytoplasmic membrane-coated PLGA nanoparticles (PM2-PLGA-NOR) were another biomimetic approach. The design also takes advantage of the homologous targeting of bacterial membrane components with the affinity of Gram-negative pathogens with polymyxin B to enable effective biofilm penetration. Norharmane interferes with quorum sensing by inhibiting PqsA, resulting in a marked decrease in biofilm formation. A 2-log decrease in bacterial lung burden was observed with intraperitoneal delivery of polymyxin B in vivo [146].

#### 2.3.2. *Mycobacterium tuberculosis*

Tuberculosis is one of the major infectious causes of mortality in the whole planet; the estimation of cases of multidrug-resistant TB was 390,000 in 2024 [147]. The special developmental issues of TB treatment include the intracellular niche of *M. tuberculosis* in alveolar macrophages, the waxy, impermeable cell wall, and the requirements for extended multidrug treatment.

An essential study demonstrated that the distribution of PLGA nanoparticles delivered intranasally to *M. tuberculosis*-infected mice is widespread throughout the lungs and selectively localised in alveolar macrophages, the primary host cells of the pathogen. Remarkably, infected macrophages internalise significantly more nanoparticles than uninfected cells, resulting in higher intracellular drug levels and improved targeting of the infectious agent. This preferential uptake is also independent of models of bacterial, viral and parasitic infections and of different nanomaterials. Surprisingly, loading the anti-TB drug bedaquiline into these nanoparticles increased its delivery efficiency to infected cells [148].

Frohlich et al. further highlighted importance of conventional tuberculosis therapy by demonstrating that, following oral delivery of 600 mg of rifampicin, approximately 2.3 μg/g of drug was detected in lung tissue. This low bioavailability, mainly due to the first-pass effect and activation of liver cytochrome 3A4, requires increased therapeutic doses, resulting in increased side effects and leading to antimicrobial resistance [149].

Mannose-functionalised silver nanocomposites have been developed to enhance targeted delivery of anti-tuberculosis agents to macrophages, improving intracellular drug action against *Mycobacterium tuberculosis*. These systems exhibit strong antimicrobial efficacy, with minimum inhibitory concentrations reduced to 16 µg/mL against MDR strains and 64 µg/mL against H37Rv, indicating enhanced therapeutic potency. Additionally, the nanocomposites maintain >90% cell viability at concentrations up to 100 µg/mL, demonstrating favourable biocompatibility alongside sustained antibacterial activity [150].

#### 2.3.3. *Streptococcus pneumoniae*

*S. pneumoniae* is a leading cause of community-acquired pneumonia, with increasing antibiotic resistance posing a major challenge [151].

AuNPs conjugated with vancomycin were created to increase antibacterial activity and inhibit colonisation of *S. pneumoniae*. The size of the nanoformulation was 23 ± 1 nm in a uniform sphere. The minimum concentration tests showed that the concentration of AuNPs (512–32 μg/mL) and vancomycin (0.5–0.125 μg/mL) required was significantly lower than when each was used alone. The nano-antibiotic is also effective in preventing bacterial adhesion and invasion in human alveolar epithelial cells (A549). Toxicity assays verified that the formulation was non-toxic to A549 cells. These findings demonstrate the potential of AuNP-mediated antibiotic delivery to enhance antibacterial activity against *S.pneumoniae* [152].

A multifunctional mucus-penetrating nanomotor system based on poly-L-arginine-modified ZIF-8 metal–organic frameworks loaded with porphyrin IX was developed for eradication of *S. pneumoniae* colonising the nasal mucosa. Upon stimulation with ultrasound, nanoplatform combined chemotherapy, sonodynamic therapy and gas therapy to enhance antibacterial activity and facilitate penetration through mucus barrier. Furthermore, the treatment generated antigenic bacterial debris that promoted in situ mucosal vaccination and immune memory, demonstrating both therapeutic and prophylactic potential against pneumococcal infection [153].

#### 2.3.4. MRSA

MRSA is a major cause of hospital-acquired and community-acquired pneumonia, with its multi-antibiotic resistance and robust biofilm-forming capacity presenting significant clinical challenges [154,155].

A copper sulfide/gold nanocluster@liposomes nanoparticles (CuS/AuNCs@Lip NPs) demonstrated potent therapeutic efficacy against MESA pneumonia in murine models. Following near-infrared irradiation, formulation effectively eradicated bacterial biofilms, accumulated preferentially at infected lung tissues and produced significant therapeutic benefits while maintaining a favorable biosafety profile [137].

Glycosylated mesoporous polydopamine nanoaerosols (MPDA/B@M) were developed to deliver bufalin selectively to pulmonary lesions through macrophage-targeted delivery. The nanoaerosol demonstrated 98.2% antibacterial activity against MRSA in vitro and a significant decrease in pulmonary bacterial loads in MRSA-infected murine pneumonia models [156].

A dual-targeted, multifunctional nanoparticle (BDPV), consisting of vancomycin-conjugated DSPE-PEG2000 encapsulating a BODIPy derivative, demonstrated excellent therapeutic efficacy against MRSA infections. The formulation effectively treated both pneumonia and skin wound infection models, illustrating the versatility of multifunctional nanoplatforms for managing MRSA-associated infections [157]. Representative nanotechnology-based antimicrobial systems and their functional outcomes are summarised in Table 2.

**Table 2:** Nanotechnology-based antimicrobial systems targeting respiratory pathogens and biofilm-associated infections.

Type/Strategy	Formulation	Target Pathogen	Key Outcomes	Therapeutic Implication	Reference
Biogenic inorganic NP	Selenium nanoparticles (Se-NPs) from *B. pumilus* and *P. aeruginosa*	*E. coli*, *P. aeruginosa*, *S. aureus*, *E. faecalis*	Smaller particle size (64 nm vs. 146 nm) correlated with enhanced antibacterial activity; up to 4-fold MIC reduction and improved biofilm eradication	Promising alternative to conventional antibiotics with enhanced antibiofilm activity	[158]
Green synthesised metallic NP	Ursolic acid-mediated AgNPs	*B. cereus*, *P. aeruginosa*, *S. aureus*, *E. coli*, *K. pneumoniae*, *E. faecalis*	Strong antimicrobial activity (ZOI up to 18 mm), low MIC, and >60% biofilm inhibition, induces membrane damage and cellular leakage	Multifunctional nanotherapeutic with antibacterial and antibiofilm potential	[159]
Hybrid polymer-metal NP	pH-responsive chitosan–Ag nanoparticles loaded with ciprofloxacin	MDR *P. aeruginosa*, *K. pneumoniae*	4-fold MIC reduction, strong biofilm inhibition (65–70%), optimised inhalation properties (MMAD 2.6 μm), synergistic activity (FICI = 0.5)	Inhalable nanoplatform for targeted pulmonary delivery against MDR infection	[160]
Metal–polyphenol NP	Gallium quercetin nanoparticles (GEQ NPs)	*P. aeruginosa*, MRSA	Disrupted ETC (reduced 83.3% enzyme activity), reduced biofilm biomass to 9.7%, and 4-log bacterial reduction in vivo	Dual mechanism therapy targeting metabolism and signalling pathways in biofilm	[145]
Bimetallic NP	Pt@Ag core–shell nanoparticles	*E. coli*, *P. aeruginosa*, *S. auerus*, Fungi	Low MIC (3.9–15.6 μg/mL), up to 95% biofilm inhibition, antifungal and antioxidant activity, high hemocompatibility	Broad-spectrum multifunctional nanoplatform for co-infections and AMR	[161]

AgNP, Silver nanoparticles; AMR, Antimicrobial resistance; ETC, Electron transport chain; FICI, Fractional inhibitory concentration indices; MDR, Multidrug resistance; MIC, Minimum inhibitory concentration; MMAD, Mass median aerodynamic diameter; MRSA, Methicillin-resistant *Staphylococcus aureus*; Pt@Ag, Platinum silver; Se-NPs, Selenium Nanoparticles; ZOI, Zone of inhibition.

## 3. Challenges and Future Direction

A critical review must also address the limitations and obstacles that impede clinical translation, as well as emerging opportunities for next-generation nanotherapeutics.

### 3.1. Limitations

Long-term safety and toxicity. Despite potent antimicrobial activity, the chronic toxicity of inhaled nanoparticles remains poorly characterised. The issue of bacterial adaptation, cytotoxicity, and non-specific interactions has led to a large body of research on innovative delivery systems to maximise the efficiency of nanoparticles and reduce their adverse effects [159]. Inhaled nanoparticles in the lung can be deposited deep in the alveolar region and retained over time, resulting in bioaccumulation in lung tissue and even translocation to extra-lung tissues, such as the kidney [162]. This build-up is linked to chronic inflammatory reactions and tissue damage, casting doubt on the long-term safety of non-biodegradable nanomaterials [163]. According to a scoping review of metal-containing nanoparticles, the formation of reactive oxygen species and the induction of inflammatory cascades, resulting in the disruption of the respiratory epithelial barrier, translocation to the cardiovascular and nervous systems, and dose-dependent genotoxicity, such as DNA strand breaks and micronuclei formation, are the major mechanisms of toxicity [164]. A biokinetic study on inhaled silver, gold, copper oxide and zinc oxide nanoparticles has shown that silver and gold were trapped in the lung longer (>2000 and >672 h, respectively) and both were accumulated in the brain and olfactory bulb, with silver remaining in the liver and the spleen 2000 h later after exposure [165]. Conversely, recent advances in nanomaterial engineering have demonstrated that biocompatibility can be substantially improved by using naturally derived materials. Tea polyphenol nanoparticles (TPNs), synthesized from epigallocatechin gallate (EGCG), exhibited excellent in vivo biocompatibility while preserving therapeutic efficacy, improving liver and kidney function, and enabling stimuli-responsive intracellular drug delivery. These findings suggest that rational material selection and surface engineering can mitigate toxicity concerns and support the safe clinical translation of next-generation nanomedicines [81].

Effects on healthy organisms. In addition to toxicity studies, several investigations have evaluated the biocompatibility of nanocarriers in healthy animals. Lipid-based and biodegradable polymeric nanoparticles generally demonstrate favourable safety profiles following pulmonary administration, producing minimal histopathological alterations in lung tissue and no significant changes in liver or kidney function at therapeutically relevant doses. Similarly, inhaled liposomal formulations have shown good local tolerability and limited systemic exposure because drug release occurs primarily within the lungs. Nevertheless, the safety profile varies considerably according to nanoparticle composition, size, surface chemistry, dose, and duration of exposure. In contrast to biodegradable lipid and polymeric systems, persistent inorganic nanoparticles may accumulate in tissues following repeated administration, warranting long-term evaluation of biodistribution, clearance, immunogenicity, and organ-specific toxicity in healthy animal models before clinical application.

Scalability and manufacturing. Nanomedicine translation to the clinic is achieved by reproducible, cost-effective and Good Manufacturing Practice (GMP)-compliant production. Control of batch-to-batch variation in particle sizes, throughput, yield and scalability is a major challenge [166]. Sterilisation of nanoparticle formulations without altering their physicochemical properties—especially for inhaled products—poses additional challenges [167].

Regulatory pathways. Nanomedicine regulatory frameworks are still developing. A review on inhalable nanomedicines for pulmonary infectious diseases notes a notable gap in translational research, with challenges in achieving the target product profile, the availability of appropriate in vivo disease models, and scale-up and market-related questions likely hindering translation to the clinic [168]. A scaling nanopharmaceutical production review reports regulatory differences across jurisdictions as a major impediment to global manufacturers aiming to increase production without compromising compliance with various standards, and the importance of GMP and quality-by-design strategies in assuring batch-to-batch consistency [169,170]. Moreover, immunogenicity, protein stability, and regulatory licensing are also major barriers to clinical translation [171].

Clinical translation and pharmaceutical adoption. Although numerous nanomaterial-based antimicrobial systems have demonstrated promising efficacy in preclinical studies, their successful translation into pharmaceutical practice remains limited. This discrepancy reflects the complexity of developing nanomedicines compared with conventional antimicrobial formulations. In addition to demonstrating superior therapeutic efficacy, nanomedicines must establish reproducible manufacturing processes, long-term physicochemical stability, scalable Good Manufacturing Practice (GMP)-compliant production, and well-defined quality attributes. Furthermore, clinical translation requires comprehensive pharmacokinetic, biodistribution, immunogenicity, and repeated-dose safety evaluations, which substantially increase development time and cost. These challenges are particularly relevant for inhaled formulations, where nanoparticle stability during aerosolization and consistent lung deposition must also be demonstrated. Consequently, despite encouraging laboratory findings, only a limited number of antimicrobial nanomedicines have received regulatory approval, highlighting the need for standardized manufacturing strategies, harmonized regulatory guidelines, and robust clinical evidence to facilitate their integration into routine pharmaceutical practice [172].

Bacterial acclimatisation towards nanoparticles. Emerging evidence indicates that bacteria can become tolerant or resistant to nanomaterials. A study of silver nanoparticle resistance in Acinetobacter baumannii found that resistant bacteria increased outer membrane proteins, extracellular polymeric substance production, oxidative stress management systems (ROS scavenger enzymes), and opportunistic metal efflux pump defence mechanisms, which were not observed in the wild-type strain [173]. This underscores the need for responsible deployment and antimicrobial stewardship for nanomedicines.

### 3.2. Future Directions

Despite these limitations, a number of new strategies are improving this area of research. Smart nanotherapeutics that incorporate stimuli-responsive elements, such as acidic pH, elevated reactive oxygen species, and bacterial enzymes, provide spatiotemporal control of drug delivery. Advances in biomineralized nanocarriers have further demonstrated that metal-carbonate nanoparticles can achieve high macromolecular drug-loading efficiency while enabling pH-responsive cargo release, illustrating the potential of biomineralization strategies for the development of next-generation antimicrobial delivery systems [76,174].

Inhalable nanomedicine formulations are also a viable step towards clinical use. In a review on inhalable nanoparticles as lung therapies, it is emphasised that the development of stimuli-responsive nanoparticles and smart inhalation devices is a promising solution, but will not achieve successful clinical translation without interdisciplinary strategies that include materials science, pharmacology and toxicology, pulmonology, and regulatory science [175].

Nanomedicine that personalises nanoparticle characteristics to patient-specific factors might be the best way to optimise therapeutic efficacy. Advances in biomineralization have also enabled the development of highly efficient metal carbonate-based nanocarriers with exceptional macromolecular loading capacity and stimuli-responsive release characteristics, providing a promising platform for future antimicrobial nanomedicine design [176]. The development of nanopharmaceuticals on a large scale is a significant issue in translating personalised nanomedicine into clinical practice. Nanoparticles based on biomimetic and immunomodulatory properties are also under development. A neutrophil membrane-encapsulated nanosonosensitizer inhibited biofilms and prevented pulmonary injury in a murine pneumonia model [144]. A study on maleimide-modified copper indium selenide nanoparticles showed that, following intranasal administration, it quickly penetrates the mucus layer and targets *P. aeruginosa* by attaching to its biofilm, which achieves bacteria inactivation and elimination of biofilm under near-infrared-II light irradiation, while also downregulating NF-κβ to reduce inflammatory cytokines and promote polarisation of macrophages toward an anti-inflammatory phenotype [121].

## 4. Conclusions

Antimicrobial resistance in respiratory infections reflects not only microbial evolution, but also the inability of conventional drug delivery to overcome the complex biological barriers of the pulmonary environment. Mucus hypersecretion, biofilm formation, intracellular persistence, and unfavourable pharmacokinetics all contribute to therapeutic failure, even when active antimicrobial agents remain available.

In this context, nanotechnology offers a delivery-oriented strategy to improve the performance of existing therapies. Lipid-based, polymeric, and inorganic nanocarriers can be engineered to enhance pulmonary deposition, improve mucus and biofilm penetration, facilitate intracellular targeting, and provide controlled or stimuli-responsive drug release. In addition, selected nanomaterials may contribute intrinsic antimicrobial effects through mechanisms such as reactive oxygen species generation, metal ion release, photothermal activity, or nitric oxide delivery. Collectively, these properties expand the range of therapeutic options available for difficult-to-treat respiratory infections.

However, the current evidence base remains weighted toward preclinical research. Although selected systems, such as liposomal amikacin for pulmonary infection, demonstrate that clinical translation is feasible, most nanotechnology-based platforms remain limited by unresolved concerns regarding long-term safety, reproducible large-scale manufacturing, regulatory complexity, and the potential for microbial adaptation. These barriers are particularly important in pulmonary applications, where repeated exposure and tissue retention may have significant biological consequences.

## Data Availability

No new data were created or analyzed in this study. Data sharing is not applicable to this article.

## Abbreviations

The following abbreviations are used in this manuscript:

AMR	Antimicrobial Resistance
MDR	Multidrug-Resistant
XDR	Extensively Drug-Resistant
WHO	World Health Organization
RTIs	Respiratory Tract Infections
CF	Cystic Fibrosis
COPD	Chronic Obstructive Pulmonary Disease
EPS	Extracellular Polymeric Substances
MIC	Minimum Inhibitory Concentration
TB	Tuberculosis
FDA	Food and Drug Administration
AWaRe	Access, Watch, Reserve Classification
PEG	Polyethylene Glycol
PEGylation	Polyethylene Glycol Surface Modification
MPS	Mononuclear Phagocyte System
MAC	Mycobacterium avium Complex
LNPs	Lipid Nanoparticles
siRNA	Small Interfering Ribonucleic Acid
TNF-α	Tumor Necrosis Factor Alpha
SLNs	Solid Lipid Nanoparticles
NLCs	Nanostructured Lipid Carriers
PLGA	Poly(lactic-co-glycolic acid)
DA-AZI NPs	Dopamine-Azithromycin Nanoparticles
MNPs	Macrophage Membrane-Coated Nanoparticles
AgNPs	Silver Nanoparticles
AuNPs	Gold Nanoparticles
ZnO NPs	Zinc Oxide Nanoparticles
CuO NPs	Copper Oxide Nanoparticles
ROS	Reactive Oxygen Species
QDs	Quantum Dots
GO	Graphene oxide
CNTs	Carbon nanotubes
MRSA	Methicillin-Resistant Staphylococcus aureus
ATP	Adenosine Triphosphate
DNA	Deoxyribonucleic Acid
RNA	Ribonucleic Acid
PDI	Polydispersity Index
